# Clinical features of androgen abuse withdrawal in men during the first year of cessation: a community dwelling study

**DOI:** 10.1210/clinem/dgag110

**Published:** 2026-03-12

**Authors:** Bonnie Grant, Joseph Kean, Nipun Lakshitha de Silva, Rodrigo Aguilera, Oliver C B Quinton, Maha Gumssani, Paul Bassett, Waljit S Dhillo, Anne Lingford-Hughes, Kim Wolff, Channa N Jayasena

**Affiliations:** Section of Investigative Medicine, Department of Metabolism, Digestion and Reproduction, Imperial College London, London W12 0NN, UK; BloodWorks Ltd, Bradford BD17 5RT, UK; Section of Investigative Medicine, Department of Metabolism, Digestion and Reproduction, Imperial College London, London W12 0NN, UK; Department of Clinical Sciences, Faculty of Medicine, General Sir John Kotelawala Defence University, Ratmalana 10370, Sri Lanka; Drug Control Centre, King's Forensics, Faculty of Life Science and Medicine, King's College London, London SE1 9NH, UK; Section of Investigative Medicine, Department of Metabolism, Digestion and Reproduction, Imperial College London, London W12 0NN, UK; Section of Investigative Medicine, Department of Metabolism, Digestion and Reproduction, Imperial College London, London W12 0NN, UK; Statsconsultancy Ltd, Bucks HP7 9EN, UK; Section of Investigative Medicine, Department of Metabolism, Digestion and Reproduction, Imperial College London, London W12 0NN, UK; Division of Psychiatry, Department of Brain Sciences, Imperial College London, London W12 0NN, UK; Drug Control Centre, King's Forensics, Faculty of Life Science and Medicine, King's College London, London SE1 9NH, UK; Section of Investigative Medicine, Department of Metabolism, Digestion and Reproduction, Imperial College London, London W12 0NN, UK

**Keywords:** androgen abuse, anabolic-androgenic steroids, testosterone, hypogonadism, erectile dysfunction, depression, withdrawal

## Abstract

**Context:**

Androgen abuse is an increasing public health concern, particularly among young men seeking muscular or image enhancement. Although most achieve biochemical recovery within 12 months of cessation, mood disturbances, sexual dysfunction, and fatigue are widely reported for years afterward.

**Objective:**

To examine symptom severity the relationship to biochemical recovery during the first year after androgen abuse cessation.

**Methods:**

We conducted a cross-sectional study of community-dwelling men grouped as nonusers, current users, or past users who had ceased within the last 12 months. Participants completed questionnaires on androgen and substance use and 4 validated instruments assessing mood (Beck Depression Inventory-II), anxiety (Generalized Anxiety Disorder-7), sexual function (International Index of Erectile Function-15), and quality-of-life (36-item Short Form). Morning fasted serum hormonal analysis and screening to exclude undisclosed androgen use were performed.

**Results:**

Two hundred forty-seven men were included: 50 nonusers, 125 current users, and 72 past users. Self-reported psychiatric diagnoses were 2.5-fold higher among current and past users than nonusers. Total testosterone (TT) was highest in current users (*P* < .001) with no difference between past and nonusers. Past users reported significantly worse mood, anxiety, sexual function, and quality of life vs nonusers. Multivariable analyses showed psychiatric comorbidity was independently associated with depression (*P* = .001), anxiety (*P* < .001), and poorer quality-of-life (*P* < .05). Older age and higher luteinizing hormone were associated with reduced sexual function (*P* < .05), while lower testosterone showed only a modest association with depressive symptoms (*P* = .03).

**Conclusion:**

Among men in the first year after stopping androgen abuse, psychological symptoms and reduced quality-of-life were most strongly associated with psychiatric comorbidity than biochemical recovery. These findings challenge the assumption that androgen withdrawal symptoms are predominantly driven by hypogonadism and supports developing psychological and behavioral interventions, rather than pharmacological, to support androgen withdrawal in men.

Androgen abuse is an increasing public health concern, particularly among young men. Previously confined within bodybuilding competitions and doping in elite sporting performance, the primary motivations for androgen abuse have shifted to image and muscular enhancement within the general male population (1, 2). A 2014 meta-analysis estimated a worldwide lifetime prevalence rate of androgen abuse in men of 6.4%, and 18.4% in recreational sportsmen (3). Androgen abuse has been associated with a 3-fold increase in mortality, primarily due to cardiovascular disease, and significant morbidity including dyslipidemia, hepatotoxicity, subfertility, and mood disorders (4-8). Population-based studies have also observed that men who tested positive for androgens had increased use of psychopharmacological treatments in the years following a doping sanction (8). Recent UK health strategies have prioritized men's mental health and suicide prevention, which are particularly prevalent among men who abuse androgens (8-10). The Centre for Social Justice has additionally reported a concerning trend in increasing androgen abuse among adolescents, indicating a growing future public health challenge (11).

Suppression of the hypothalamic–pituitary–gonadal (HPG) axis occurs with androgen abuse due to the negative feedback from exogenous androgens on hypothalamic gonadotrophin-releasing hormone and pituitary gonadotrophin (luteinizing hormone [LH] and follicle-stimulating hormone [FSH]). Recent high-quality studies suggest that biochemical gonadal recovery occurs in most men within 12 months of cessation, with factors such as duration and intensity of androgen abuse influencing time to recovery (4, 12-15). The nature and severity of clinical symptoms and the relationship with biochemical recovery during the first year of cessation are not known.

Many men experience withdrawal symptoms of sexual dysfunction, fatigue, and de novo or worsening mood disturbances with androgen abuse cessation (16). Existing studies have described the range of symptoms reported by men who discontinued androgen abuse several months to many years earlier, in some cases up to a decade prior (13, 14, 17). Although symptoms have been reported to persist for many years in some individuals, currently no study has focused on the first year after cessation. This is a clinically critical window when the reproductive axis is recovering and when men are most likely to present for clinical assessment of symptoms. The absence of data in this early phase may explain why prior studies have failed to identity associations between serum testosterone and symptoms of sexual or mood disturbance (13, 14, 18). Additionally, biochemical verification of androgen abstinence is inconsistently used in the current literature. Understanding this early period and the relationship between androgen withdrawal symptoms and serum testosterone levels is crucial, as men experiencing severe withdrawal symptoms are at highest risk of relapse, with concerns about testosterone recovery commonly cited as a barrier to cessation (16, 19). Prior studies have reported that ∼65% of men restart androgen abuse more than 1 year following cessation, particularly those with higher cumulative exposure (19). In other substance use disorders, relapse rates have been reported as high as 70% within the first year following cessation, with withdrawal symptom severity, socioeconomic factors, poor physical, and psychiatric health shown to predict substance use relapse (20). Understanding the first year following androgen abuse cessation is therefore important for clinical assessment, targeted intervention, and relapse prevention.

To address these gaps, our study is the first to examine the relationship between symptoms and biochemical recovery during the first 12 months following self-reported cessation of androgen abuse, confirmed by urinary testing to exclude undisclosed androgen consumption, with the aim of characterizing the spectrum of symptoms and identifying factors associated with symptom severity. This information will help assess the potential for androgenic pharmacological therapies as part of an evidenced-based treatment regime, to ultimately support cessation and prevent relapse of androgen abuse.

## Materials and methods

### Study design

We conducted a cross-sectional, observational study of community-dwelling male participants. Recruitment occurred from May 2023 to July 2024 across 4 locations in the United Kingdom: London, West Yorkshire, Bristol, and Devon. The study received ethical approval from London—Fulham Research Ethics Committee on March 31, 2023 (23/LO/0124). The study was performed in accordance with the Declaration of Helsinki. All participants provided written, informed consent. In the United Kingdom, androgens are a controlled substance, classified as a Class C substance under Part III of Schedule 2 of the Misuse of Drugs Act 1971 and Schedule 4 of the Misuse of Drugs Regulation 2001 and possession for personal use is not considered an offense.

### Participants

**Figure 1:**
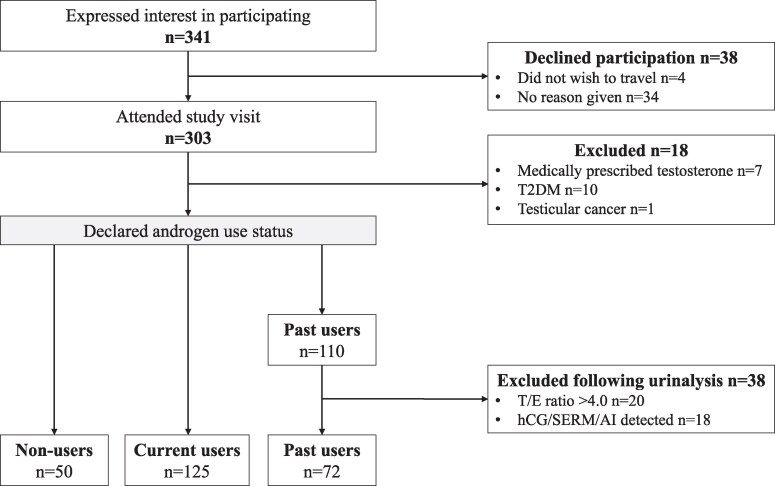
Flow diagram of participant inclusion and exclusion. AIs, aromatase inhibitors; hCG, human chorionic gonadotrophin; SERM, selective estrogen receptor modulators; T/E ratio, testosterone/epitestosterone ratio.

Men aged 18 to 60 years old were recruited initially into 1 of 3 groups based on self-declaration: (1) nonandrogen use, nonusers (2) current androgen use, current users; and (3) androgen use within the past 12 months, past users. Men with any cause of organic hypogonadism other than androgen abuse, medically prescribed testosterone therapy, or systemic comorbidities likely to impact HPG axis function such as type 2 diabetes or obesity were excluded (Fig. 1). The study was advertised via the NIHR Imperial College Research Facility, on social media, online fitness and bodybuilding forums, posters in gyms and via The Anabolic Steroid UK network of harm practitioners. Many of these practitioners work within needle and syringe programs (NSPs) based within alcohol and drug services across the United Kingdom and recruited participants via word-of-mouth and directed local advertising.

### Study procedure

During a single, fasted, morning visit (before 10:30 Am), participants completed a standardized questionnaire of demographic information, self-reported medical and psychiatric comorbidities, history of androgen and other substance misuse and 4 validated patient reported outcome questionnaires. Measurements were taken for height, weight, and blood pressure and blood and urine samples were collected.

### Validated symptom questionnaires

Study participants completed 4 validated patient reported outcome questionnaires. The Beck Depression Inventory-II (BDI-II) is a 21-question self-reported measure of depressive symptoms with a score ranging from 0 to 63 (21). Depression severity can be graded as: 0 to 13 minimal depression; 14 to 19 mild depression; 20 to 28 moderate depression; 29 to 63 severe depression. The Generalized Anxiety Disorder-7 (GAD-7) is a 7-question self-reported measure of symptoms of anxiety with a total score ranging from 0 to 21 (22). Anxiety severity can be graded as follows: 0 to 4 minimal anxiety; 5 to 9 mild anxiety; 10 to 14 moderate anxiety; 15 to 21 severe anxiety. The International Index of Erectile Function-15 (IIEF-15) is a 15-question self-administered questionnaire of sexual function (23). The total score ranges from 6 to 75 with 5 subdomains of erectile function, orgasmic function, sexual desire, intercourse satisfaction, and overall satisfaction, which have a maximal score of 30, 10, 10, 15, and 10 respectively. Lower scores are indicative of sexual dysfunction. The 36-item Short-Form (SF-36) Survey is used as a measure of health status and quality of life (QOL) (24). This self-administered questionnaire of 36 questions measures 8 domains of health: physical functioning; role limitations due to physical health; role limitations due to emotional problems; energy and fatigue; emotional wellbeing; social functioning; pain; and general health. Each domain has a score ranging from 0 to 100, with lower scores suggestive of poorer health status. Using a predefined formula, mental and physical component scores (MCS and PCS) are calculated from *Z*-scores using mean values and standard deviations (SDs) from the US general population for each of the 8 SF-36 domains (25). It has previously been shown that these calculated means and SDs can be used as standard across different population groups, including within the UK population (26).

### Laboratory analysis

Blood samples were taken for total testosterone (TT), LH, FSH, sex hormone–binding globulin (SHBG), 17β-estradiol (E2), hemoglobin, hematocrit, glycated hemoglobin (HbA1c), total cholesterol (TC), low-density lipoprotein (LDL) cholesterol, high-density lipoprotein (HDL) cholesterol, triglycerides, albumin, bilirubin, alanine transaminase (ALT), and alkaline phosphatase (ALP). TT, LH, FSH, SHBG, E2, TC, LDL, HDL, triglycerides, albumin, bilirubin, ALT, and ALP assays were performed on the Abbott ALINITY system in Charing Cross Hospital, Imperial College Healthcare NHS Trust with an assay coefficient of variation (CV) <10%. Regular internal quality checks are undertaken in the laboratory, and it is subject to UK National External Quality Assurance Service monitoring. Hemoglobin and hematocrit samples were analyzed on the point-of-care Hemo Control Hemoglobin analyzer with a CV <2% (27). HbA1c was analyzed on the point-of-care HemoCue® HbA1c 501 System with a CV 3.4% (28).

### Urine analysis

Urine samples were collected in a 70-mL sterile specimen container by participants. Samples were stored at −20 °C until analysis, performed by the Drug Control Centre, King's College London. The Drug Control Centre is the only World Anti-Doping Agency (WADA) UKAS accredited laboratory in the United Kingdom. Analysis was performed on all samples of men declaring past-use. Measurements were performed in line with the WADA technical document TD2021EAAS for measuring and reporting endogenous anabolic-androgenic steroid markers of the urinary steroid profile (29). Concentrations of testosterone (T) and epitestosterone (E) was determined by gas chromatography-mass spectrometry (MS) from the combination of the free steroid fraction and the conjugated fraction, released after hydrolysis with β-glucuronidase from *Escherichia coli*. The T/E ratio was calculated from these concentrations. The limits of quantification (LOQ) for T and E were ≤1 ng/mL, with a measurement uncertainty at LOQ of <30% (29). A T/E cutoff of >4.0 was used to exclude men as suspicious for nondisclosed androgen abuse, in line with WADA recommendations. The presence of selective estrogen receptor modulators (SERMs) and aromatase inhibitors (AIs) was identified liquid chromatography-MS to detect parent compounds and metabolites, while human chorionic gonadotrophin (hCG) is detected via immunoassay, with concentrations >5 IU/L suspicious for use (30). Twenty men who reported prior use were found to have a urinary T/E ratio >4.0, and a further 18 were found to have urinary evidence of hCG, SERMs, or AI use and were excluded (Fig. 1).

### Data analysis

Statistical analysis was performed using GraphPad Prism Version 10.0. Continuous variables are presented as mean ± SD or median with interquartile range. Normally distributed data was analyzed with unpaired t-test with Welch's correction or 1-way analysis of variance (ANOVA) as appropriate. Nonnormally distributed data was analyzed with Mann–Whitney test or Kruskal–Wallis test with Dunn's test. Categorical variables are presented as absolute numbers and percentages and analyzed by chi-square or Fisher's exact test. *P*-values for linear contrasts between groups are only shown when the overall ANOVA showed significant differences. In past users, inverse prediction from linear regression models was used to estimate the time since cessation required to achieve the mean nonuser concentrations of TT, LH, and FSH. Spearman's correlation analysis was performed for the entire cohort to assess associations between serum TT and estradiol concentrations and symptom scores. Where multiple comparisons were made, *P*-values were adjusted using the Bonferroni method. Multiple linear regression analysis was performed using Stata version 19 for the past users for the 5 key symptom scores (BDI-II, GAD7, IIEF-15, SF36 PCS, and SF36 MCS), which were the outcome variables in the analysis, measured on a continuous variable. Covariates included were age, self-reported psychiatric diagnoses, lifetime duration of use (years), time since cessation (months), hCG/SERM/AI use, illicit drug use, TT, and LH. Serum TT was analyzed as a continuous variable in the regression model, with effect estimates reflecting absolute unit changes in measured concentration rather than changes relative to baseline or age-specific reference ranges. Outcome variables found to be normally distributed were analyzed on the original scale of measurement with coefficient and confidence interval, while outcomes with a positively skewed distribution were given a log transformation before analysis and presented as ratio with confidence interval. The shape of the relationship between continuous predictors and the outcomes were examined. Where this improved the fit of the model, squared terms for each factor were included in the regression model.

## Results

### Participant characteristics

A total of 50 nonusers, 125 current users, and 72 past users with a negative urine screen for androgens, hCG, SERMs, or AIs were included in the analysis (Fig. 1). Participant characteristics are summarized in Table 1.

**Table 1:** Participant characteristics by group

	Nonusers n = 50	Current users n = 125	Past users n = 72	*P*-value (nonusers vs current users)	*P*-value (nonusers vs past users)	*P*-value (current users vs past users)
**Demographics**						
Age (years)	36.9 ± 11.4	36.3 ± 9.0	36.6 ± 8.9			
Ethnicity						
Asian or Asian British	11 (22.0)	18 (14.4)	16 (22.2)			
Black, Black British, Caribbean, or African	1 (2.0)	4 (3.2)	0 (0.0)			
Mixed or multiple ethnic groups	3 (6.0)	5 (4.0)	4 (5.6)			
Other ethnic group	4 (8.0)	2 (1.6)	0 (0.0)			
White	31 (62.0)	96 (76.8)	52 (72.2)			
Psychiatric diagnosis	5 (10.0)	34 (27.2)	15 (20.8)	.015		
Anxiety	3 (6.0)	22 (17.6)	12 (16.7)			
Body dysmorphia	0 (0.0)	3 (2.4)	0 (0.0)			
Depression	5 (10.0)	27 (21.6)	12 (16.7)			
Eating disorder	0 (0.0)	1 (0.8)	0 (0.0)			
History of suicidal attempt	0 (0.0)	7 (5.6)	2 (2.8)			
Other	1 (2.0)	4 (3.2)	0 (0.0)			
Alcohol use (>6 units per week)	19 (38.0)	40 (32.0)	35 (48.6)			
Nicotine use	12 (24.0)	44 (35.2)	33 (45.8)		.02	
Illicit substance use	11 (22.0)	54 (43.2)	31 (43.1)	.01	.02	
Polysubstance use	7 (14.0)	24 (19.2)	11 (15.3)			
Type of substance use						
Amphetamines	0 (0.0)	1 (0.8)	3 (4.2)			
Benzodiazepines	1 (2.0)	14 (11.2)	4 (5.6)			
Cannabis	9 (18.0)	28 (22.4)	21 (29.2)			
Cocaine	5 (10.0)	36 (28.8)	16 (22.2)			
Heroin	0 (0.0)	1 (0.8)	0 (0.0)			
Ketamine	3 (6.0)	4 (3.2)	3 (4.2)			
LSD	2 (4.0)	3 (2.4)	1 (1.4)			
MDMA/ecstasy	4 (8.0)	14 (11.2)	4 (5.6)			
Opioids (excluding heroin)	1 (2.0)	9 (7.2)	5 (6.9)			
**Clinical measurements**						
BMI (kg/m^2^)	24.8 ± 3.5	30.1 ± 3.6	29.6 ± 5.6	<.001	<.001	
Systolic BP (mmHg)	127 ± 15	146 ± 19	139 ± 17	<.001	<.001	.038
Diastolic BP (mmHg)	79 ± 10	82 ± 16	85 ± 13			
**Androgen use characteristics**						
Age first use (years)	—	28.4 ± 7.9	29.0 ± 7.1			
Lifetime duration of use (years)	—	7.9 ± 7.22	8.2 ± 8.4			
Recent cycle length	—					
<3 months		56 (44.8)	42 (58.3)			
3-6 months		39 (31.2)	16 (22.2)			
6-12 months		13 (10.4)	8 (11.1)			
>12 months		17 (13.6)	6 (8.3)			
Number of androgens used	—	2.0 (2.0, 4.0)	2.0 (1.0, 3.0)			
Type of androgens used	—					
Testosterone esters		123 (98.4)	54 (75.0)			
19-Nortestosterone		56 (44.8)	25 (34.7)			
17α-alkylated		42 (33.6)	27 (37.5)			
Other synthetic androgens		61 (48.8)	19 (26.4)			
Weekly testosterone enantate dose (mg)		325 (250, 500)	300 (250, 500)			
Use of hCG/SERM/AI	—	49 (39.2)	31 (43.1)			
Use of other IPEDs	—	50 (40.0)	23 (31.9)			
Clenbuterol		32 (25.6)	16 (22.2)			
Growth hormone		20 (16.0)	7 (9.7)			
Insulin		10 (8.0)	0 (0.0)			
Melanotan		6 (4.8)	4 (5.6)			
Selective androgen receptor modulators		1 (0.8)	3 (4.2)			
Thyroid hormones		12 (9.6)	0 (0.0)			
Time since cessation (months)	—	—	5 (2.25, 8.75)			

Values are presented as mean ± SD, median (IQR), or frequency (percentage) as appropriate. *P*-values for linear contrasts are reported only when the overall analysis of variance indicated a significant difference.

Abbreviations: AI, aromatase inhibitor; BMI, body mass index, BP, blood pressure; hCG, human chorionic gonadotrophin; IPED, image and performance enhancing drug; IQR, interquartile range; LSD, lysergic acid diethylamide; MDMA, 3,4-methylenedioxymethamphetamine; SD, standard deviation; SERM, selective estrogen receptor modulator.

Study groups did not differ significantly for age nor ethnicity. Compared with nonusers, self-reported psychiatric diagnoses were more frequently reported among current users (10% [5/50], nonusers; 27.2% [34/125], current users; *P* = .015), with no significant difference observed in past users (20.7% [15/72]). Illicit drug use was also more frequently reported among current users (22% [11/50], nonusers; 43.2% [54/125], current users; *P* = .009) and past users (22% [11/50], nonusers; 43.1% [31/72], past users; *P* = .02) compared with nonusers. Men never using androgens had lower BMIs and systolic blood pressure compared with both current users (*P* < .001) and past users (*P* < .001). Systolic blood pressure was also higher in men with current use compared with past use (*P* = .038). No significant difference was observed in diastolic blood pressure among study groups.

Among men with current or past androgen use, there were no significant differences in age at first androgen use, lifetime duration of androgen abuse, length of most recent androgen cycle, numbers of different androgens used, types of androgens used, use of hCG/SERMs, and use of other image and performance enhancing drugs. There was no difference in weekly testosterone enantate dose, the most reportedly used androgen in both groups (weekly testosterone enantate dose mg: 325 [250, 500], current users; 300 [250, 500], past users; *P* > .05). The median time since cessation in the past users was 5 months.

### Biochemical and hematological tests

Biochemical and hematological markers in each group are shown in Table 2. Compared with nonusers, serum TT concentrations were significantly higher in the current users (TT nmol/L: 20.6 ± 7.3, nonusers; 72.2 ± 37.2, current users; *P* < .001), while no significant difference was observed between nonusers and past users. Testosterone concentrations were significantly higher in current users compared with past users (TT nmol/L: 72.2 ± 37.2, current users; 14.0 ± 8.0, past users; *P* < .001). Free testosterone and estradiol concentrations were also higher in current users than both the nonusers and past users (*P* < .001).

**Table 2:** Biochemical and hematological markers by group

	Nonusers n = 50	Current users n = 125	Past users n = 72	*P*-value (nonusers vs current users)	*P*-value (nonusers vs past users)	*P*-value (current users vs past users)
**Total testosterone (nmol/L)**	20.6 ± 7.3	72.2 ± 37.2	14.0 ± 8.0	<.001		<.001
**Free testosterone (nmol/L)**	0.4 ± 0.2	3.1 ± 2.0	0.3 ± 0.2	<.001		<.001
**LH (IU/L)**	3.0 ± 1.2	0.1 ± 0.1	2.6 ± 1.4	<.001	.048	<.001
**FSH (IU/L)**	4.1 ± 3.4	0.2 ± 0.2	3.1 ± 1.8	<.001	.014	<.001
**SHBG (nmol/L)**	35.4 ± 17.0	14.5 ± 9.8	27.6 ± 12.4	<.001	.002	<.001
**Estradiol (pmol/L)**	115 ± 27	308 ± 228	103 ± 11	<.001		<.001
**Hemoglobin (g/L)**	152 ± 11	171 ± 17	158 ± 13	<.001		<.001
**Hematocrit (L/L)**	0.45 ± 0.03	0.5 ± 0.0491	0.46 ± 0.0382	<.001		<.001
**HbA1c (mmol/mol)**	34.7 ± 3.38	34.5 ± 6.99	34.9 ± 4.69			
**TC (mmol/L)**	4.8 ± 1.4	4.5 ± 1.2	4.8 ± 1.2			
**LDL-C (mmol/L)**	3.1 ± 1.2	3.1 ± 1.1	3.0 ± 1.1			
**HDL-C (mmol/L)**	1.3 ± 0.3	0.9 ± 0.3	1.2 ± 0.3	<.001		<.001
**TC/HDL Ratio**	3.7 ± 1.0	5.3 ± 2.9	4.3 ± 1.8	<.001		.005
**Triglycerides (mmol/L)**	1.0 ± 0.5	1.0 ± 0.7	1.3 ± 1.0		.046	.023
**Albumin (g/L)**	43.2 ± 4.2	38.2 ± 7.6	41.8 ± 4.7	<.001		<.001
**Bilirubin (**µmol/L)	11.7 ± 4.8	10.6 ± 5.2	10.9 ± 6.3			
**ALT (U/L)**	29.6 ± 11.9	54.8 ± 34.0	40.4 ± 20.9	<.001		.001
**ALP (U/L)**	72.2 ± 19.1	65.9 ± 21.6	77.8 ± 17.7			<.001

Data shown as mean ± SD. *P*-values for linear contrasts are reported only when the overall analysis of variance indicated a significant difference.

Abbreviations: ALP, alkaline phosphatase; ALT, alanine transaminase; FSH, follicle-stimulating hormone; HbA1c, glycated hemoglobin; HDL-C, high-density lipoprotein cholesterol; LDL-C, low-density lipoprotein cholesterol; LH, luteinizing hormone; SD, standard deviation; SHBG, sex hormone-binding globulin; TC, total cholesterol.

Serum LH and FSH concentrations were lower in the current users compared with nonusers (*P* < .001) and in past users compared with nonusers (*P* = .048). Men in the current user group had higher hemoglobin, hematocrit, TC/HDL ratio and ALT levels with lower HDL-C and albumin levels compared with the nonusers and past users (*P* < .05). In past users, the estimated time to reach mean nonuser concentrations was 12.8 months for LH and 18.9 months for FSH following androgen abuse cessation.

### Depressive symptoms

**Figure 2:**
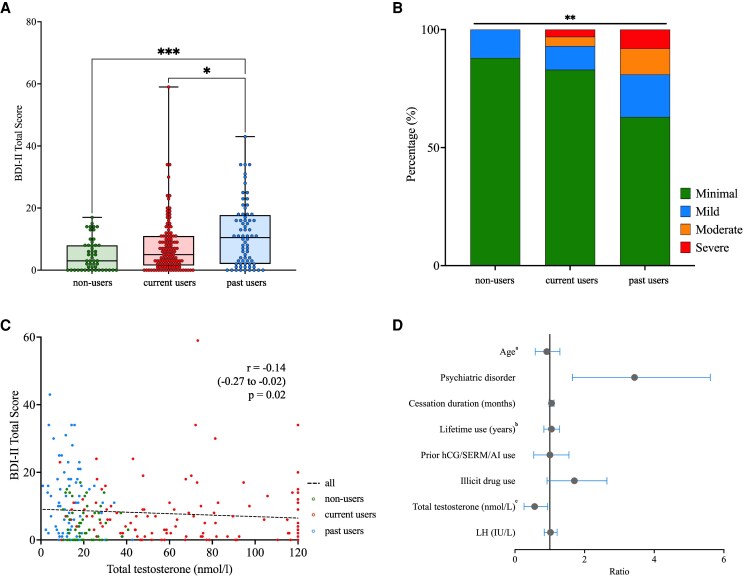
Depressive symptoms assessed by the Beck Depression Inventory-II (BDI-II). (A) BDI-II scores by group. (B) BDI-II scores by severity (0-13 minimal depression; 14-19 mild depression; 20-28 moderate depression; 29-63 severe depression). (C) Correlation between serum TT concentration (nmol/L) and BDI-II score. (D) Multivariable regression analysis of factors associated with BDI-II score in the past-use group only. *P < .05; **P < .01; ***P < .001; ^a^regression coefficient per 10-year increase in age; ^b^regression coefficient per 5-year increase in lifetime androgen use; ^c^regression coefficient per 20 nmol/L increase in total testosterone.

Median BDI-II scores were higher in past users compared with nonusers (median BDI-II: 3.0 [0.0, 8.0], nonusers; 10.5 [2.0, 17.8], past users; *P* < .001) and current users (median BDI-II: 5.0 [1.5, 11.0], current users; 10.5 [2.0, 17.8], past users; *P* = .02) (Fig. 2A). A significant difference in depression severity was observed across the groups (moderate or severe depression: 0% [0/0], nonusers; 7.2% [9/125], current users; 19.4% [14/72], past users; *P* = .004) (Fig. 2B). A weak negative correlation was observed between TT levels and BDI scores for the whole cohort (*r* = −0.14 [95% confidence interval {CI} −0.27 to −0.02]; *P* = .02) (Fig. 2C). No correlation was observed between serum TT concentrations and BDI-II scores for each group (Table S1) (31). There was no correlation between serum estradiol concentrations and BDI-II scores for the whole cohort (data not shown). Multiple linear regression was performed with BDI-II scores as the dependent outcome (Fig. 2D). A self-reported psychiatric diagnosis was associated with higher BDI-II scores (ratio for self-reported psychiatric diagnoses: 3.04 [95% CI 1.65-5.61]; *P* = .001). Total testosterone concentrations were also associated with lower BDI scores (ratio for 20 nmol/L increase in TT: 0.49 [95% CI 0.26-0.94]; *P* = .03). No significant association was observed between the other variables and BDI-II scores.

### Anxiety symptoms

**Figure 3:**
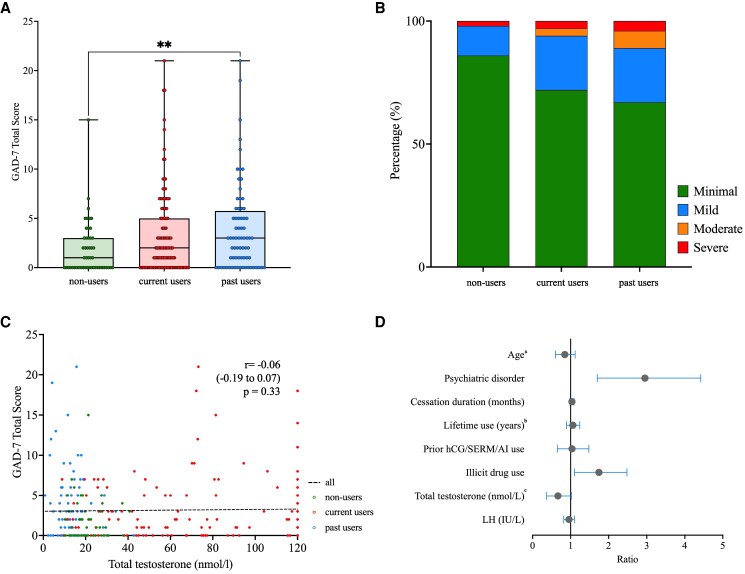
Anxiety symptoms assessed by the General Anxiety Disorder-7 (GAD-7). (A) GAD-7 scores by group. (B) GAD-7 scores by severity (0-4 minimal anxiety; 5-9 mild anxiety; 10-14 moderate anxiety; 15-21 severe anxiety). (C) Correlation between serum total testosterone concentration (nmol/L) and GAD-7 score. (D) Multivariable regression analysis of factors associated with GAD-7 score in the past-use group only. **P < .01; ^a^regression coefficient per 10-year increase in age; ^b^regression coefficient per 5-year increase in lifetime androgen use; ^c^regression coefficient per 20 nmol/L increase in total testosterone.

Median GAD-7 scores were higher in past users compared with nonusers (median GAD-7: 1.0 [0.0, 3.0], nonusers; 3.0 [0.0, 5.75], past users; *P* = .01) (Fig. 3A). No significant difference was observed in anxiety severity between the groups (Fig. 3B) nor any correlation between GAD-7 scores and TT for the whole cohort (Fig. 3C). No correlation was observed between serum TT concentrations and GAD-7 scores for each group (Table S2) (31). There was no correlation between serum estradiol concentrations and GAD-7 scores for the whole cohort (data not shown). Multiple linear regression was performed with GAD-7 scores as the dependent outcome (Fig. 3D). Self-reporting a psychiatric disorder was associated with an increase in GAD-7 scores (ratio for self-reported psychiatric diagnoses: 2.74 [95% CI 1.70-4.42]; *P* < .001). Illicit drug use was associated with an increase in GAD-7 scores (ratio for illicit drug use: 1.65 [95% CI 1.10-2.48]; *P* = .02). No association was observed with the other variables.

### Sexual function

**Figure 4:**
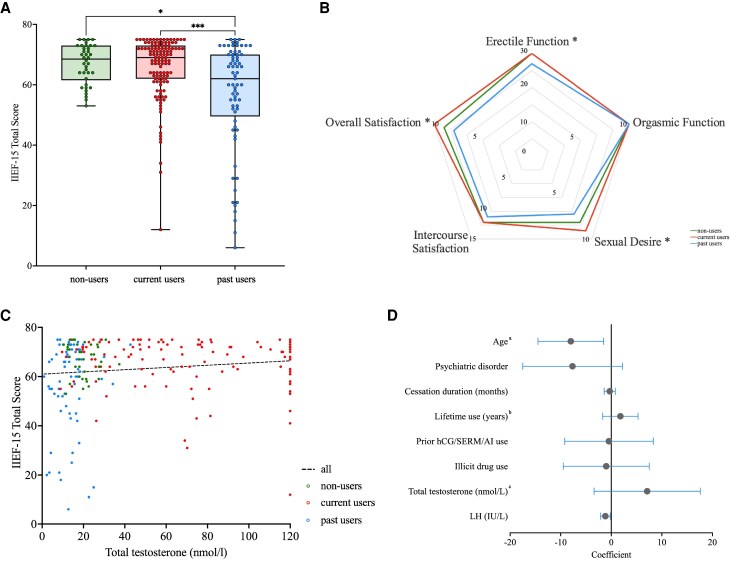
Sexual dysfunction assessed by the International Index of Erectile Function-15 (IIEF-15). (A) Total IIEF-15 scores by group. (B) IIEF-15 subdomain scores (erectile function, orgasmic function, sexual desire, intercourse satisfaction, and overall satisfaction) by group. (C) Correlation between serum total testosterone concentration (nmol/L) and total IIEF-15 score. (D) Multivariable regression analysis of factors associated with IIEF-15 score in the past-use group only. *P < .05; ***P < .001; ^a^regression coefficient per 10-year increase in age; ^b^regression coefficient per 5-year increase in lifetime androgen use; ^c^regression coefficient per 20 nmol/L increase in total testosterone.

Men with past use had lower median IIEF-15 total scores compared with nonusers (median IIEF-15 total score: 68.5 [61.5, 73.0], nonusers; 62.0 [49.5, 70.0], past users; *P* = .02) and current users (median IIEF-15 total score: 69.0 [62.0, 73.0], current users; 62.0 [49.5, 70.0], past users; *P* < .001) (Fig. 4A). Compared with nonusers, men with past use had lower IIEF-15 subdomain scores for erectile function (*P* < .001), sexual desire (*P* < .001), and overall satisfaction (*P* < .001) (Fig. 4B; Table S3) (31). Serum TT concentrations did not significantly correlate with IIEF-15 total scores (Fig. 4C); however, a positive correlation was observed with sexual desire (*r* = 0.20 [95% CI 0.07-0.33]; *P* = .012) and overall satisfaction (*r* = 0.21 [95% CI 0.08-0.34]; *P* = .006) subdomain scores of the IIEF-15 for the whole cohort (Table S4) (31). No correlation was observed between serum TT concentrations and IIEF-15 scores for each group (Table S5) (31). There was no correlation between serum estradiol concentrations and IIEF-15 total or subdomain scores for the whole cohort (data not shown). Multiple regression with IIEF-15 total score as the dependent outcome was performed (Fig. 4D). After adjustment for covariates included in the model, increasing age (β: −8.0 [95% CI −14.5 to −1.5]; *P* = .02) and serum LH levels (β: −1.2 [95% CI −2.1 to −0.2]; *P* = .01) were independently associated with lower IIEF-15 scores. The relationship between LH and IIEF-15 scores was observed to follow a nonlinear relationship, with little relationship between LH and IIEF-15 scores for LH values below 4 IU/L; however, for LH values above 4 IU/L, there were decreases in IIEF-15 scores with further increases in LH.

### Quality of life

**Figure 5:**
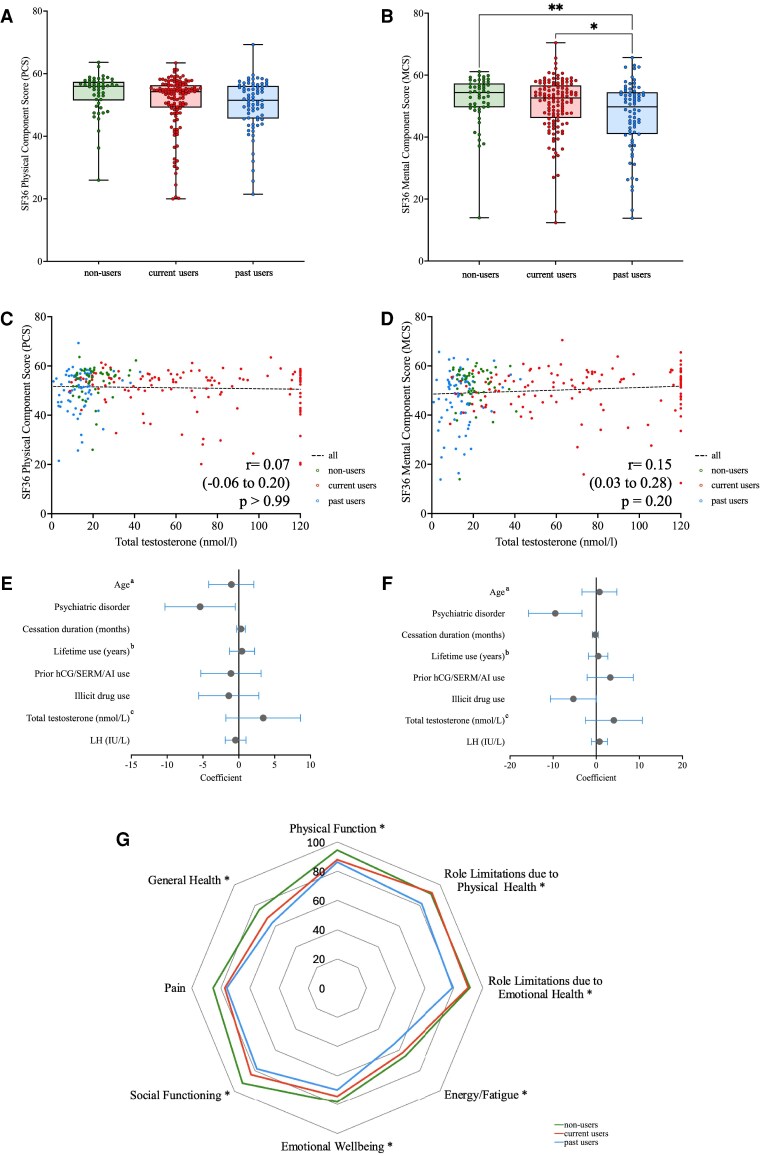
Quality of life assessed by the Short-Form-36 (SF-36). (A) SF-36 physical component scores (PCS) by group. (B) SF-36 mental component scores (MCS) by group. (C) Correlation between serum total testosterone concentration (nmol/L) and PCS. (D) Correlation between serum total testosterone concentration (nmol/L) and MCS. (E) Multivariable regression analysis of factors associated with MCS in the past-use group only. (F) Multivariable regression analysis of factors associated with MCS in the past-use group only. (G) SF-36 subdomain scores by group. *P < .05, **P < .01; ^a^regression coefficient per 10-year increase in age; ^b^regression coefficient per 5-year increase in lifetime androgen use; ^c^regression coefficient per 20 nmol/L increase in total testosterone.

Quality of life was measured using the SF-36 questionnaire with scores for each domain and physical component score (PCS) and mental component score (MCS) calculated from the responses. No significant difference in PCS was observed among the groups. Men with past use had lower MCS compared with nonusers (mean PCS: 52.0 ± 8.1, nonusers; 46.8 ± 11.3, past users, *P* = .01) and current users (mean PCS: 50.5 ± 8.9, current users, 46.8 ± 11.3, past users; *P* = .03) (Fig. 5A and 5B). No linear correlation was observed between TT levels and SF-36 PCS or MCS for the whole cohort (Fig. 5C and 5D). There was no correlation between serum estradiol concentrations and SF-36 PCS or MCS for the whole cohort (data not shown). Multivariable linear regression was performed with PCS and MCS as the dependent outcomes (Fig. 5E and 5F). Self-reporting a psychiatric disorder was associated with lower SF-36 PCS (SF-36 PCSβ: −5.4 [95% CI −10.3 to −0.5]; *P* = .03) and SF-36 MCS (SF-36 MCS β: −9.5 [95% CI −15.7 to −3.3]; *P* = .003). No other variables were associated with either SF-36 score, including serum TT concentrations. Men with past use had significantly lower scores across many of SF-36 subdomains when compared with nonusers and current users (Fig. 5G; Table S3) (31). A weak positive correlation was observed between serum TT concentrations and the SF-36 subdomain role limitation due to physical health (*P* = .016), but not with any other SF-36 subdomain for the whole cohort (Table S4) (31). A positive correlation was observed between serum TT concentrations and the SF-36 subdomain physical functioning in the nonuser group (*P* = .006), but no other correlation was observed between SF-36 subdomains and individual groups (Table S6) (31).

## Discussion

Androgen abuse is an increasing public health concern worldwide, associated with substantial physical and psychiatric morbidity (32). Up to 30% of individuals who abuse androgens may go on to develop a dependency syndrome, with 5% to 10% feeling unable to stop due to withdrawal symptoms (33-35). Fears over worsening QOL, declining muscle mass, and recovery of testosterone levels are often cited as reasons against cessation (16, 35). This is the largest study to date to comprehensively evaluate the range of symptoms experienced by men withdrawing from androgen abuse and determine independent factors associated with symptom severity. We have shown that depression, anxiety, sexual dysfunction, and QOL patient reported outcome measures are worse in men with a history of androgen abuse within the last 1 year, compared with men with current use and nonuse. Self-reported psychiatric comorbidity was shown to have the strongest association with these symptoms, with a weaker association with other illicit drug use, higher serum LH levels, and lower serum TT levels. Our findings challenge the commonly held assumption that symptoms following androgen abuse withdrawal are primarily driven by hypogonadism, highlighting the need for psychological rather than pharmacological approaches to support recovery. These results also suggest that many men may be abusing androgens to self-medicate against preexisting mood and anxiety symptoms. This has important implications for the UK's recent Men's Health Strategy, which recognizes the disproportionate rate of substance misuse in men and sets out plans to strengthen drug and alcohol treatment services, including integrated mental health and recovery support, and the Addiction Healthcare Goals program, which supports the innovation and development of new, evidence-based treatments for substance misuse (9, 36).

The findings from this study reflect real-world data of community-dwelling men across multiple geographical locations in the United Kingdom, with many of the areas visited known to have a high prevalence of androgen abuse. Men with a history of androgen abuse are often reluctant to seek traditional routes for medical attention; therefore, our study is a more accurate reflection of the wider androgen abuse community, than those referred to specialist services such as endocrine clinics (37). All blood samples were tested within a single, UKAS accredited laboratory and were taken in the morning and fasted to minimize inaccuracies of testosterone levels (38, 39). Urine samples were analyzed in the only UK UKAS accredited WADA laboratory, allowing us to exclude men who may still be exposed to high levels of exogenous androgens or other medications known to influence serum TT concentrations such as hCG and SERMs. Finally, this study focuses on the first year of cessation, which has been shown to be a high-risk period for relapse in androgen abuse, and other drugs of abuse, and a critical time for effective intervention.

However, this study is not without limitations. Due to the cross-sectional study design, causal relationships cannot be inferred, and reporter bias may have led to the inclusion of past androgen users with more severe symptoms. Participants were asked to recall information about androgens used, and duration of use, which is subject to recall bias. Detailed assessment of androgen dose was not possible due to the heterogenous nature of androgen regimes with varying potencies and the high likelihood of product contamination or mislabeling within the unregulated black market (40). Smoking, alcohol and illicit substance use intensity, dietary behaviors and cessation strategies were not assessed and may influence symptom severity. Measures of body composition were not collected, limiting assessment of physical changes during androgen abuse. Future longitudinal studies are needed to examine how these factors may influence withdrawal symptoms following androgen abuse. Although efforts were made to include multiple geographical regions, and advertisements did not specify that the study was examining symptoms, recruitment bias may have occurred for symptomatic men seeking further information. A T/E ratio above 4.0 was used to exclude possible ongoing exposure to exogenous androgens, which is in line with WADA recommendations. However, there are some instances where men may have a naturally higher T/E ratio, and we may therefore have excluded some men who did not have ongoing exogenous androgen exposure (41). Urine analysis could not be used to exclude undisclosed nontestosterone androgen abuse, given the lack of robust published data on the duration of urinary detection of these metabolites in men. Our findings highlighted the importance of psychiatric diagnoses on symptomatology, but we did not have data on whether these predated initiating androgens, occurred during use, or de novo following cessation, which should be evaluated in prospective longitudinal studies. Additionally, the presence of psychiatric diagnoses was self-reported and not verified with hospital records or medical assessment. Finally, it is worth commenting that while patient reported outcome questionnaires such as the BDI-II and IIEF-15 can be used to screen for and monitor a course of treatment, they are not diagnostic tools for sexual dysfunction and depression respectively.

The relationship between androgen abuse and psychopathology is a complex, possibly multidirectional one. Testosterone is thought to play a role in mood disorders; men with congenital hypogonadotropic hypogonadism have been reported to exhibit worse depression, anxiety, and QOL scores compared to healthy controls (42). However, these differences may not be solely attributable to low testosterone concentrations and could also reflect the psychological impact of the diagnosis or physical changes such as altered body fat distribution or gynecomastia. It is widely described that a preexisting mental health condition, such as low mood or body/muscle dysmorphia, whereby an individual views their body to be insufficiency muscular despite objective evidence otherwise, have higher rates of androgen abuse compared to men without (43, 44). Altered inflammatory pathways and neurodegeneration following supraphysiological androgen exposure may contribute to neuropsychiatric symptoms, although human evidence remains limited (45, 46). We observed that the presence of psychiatric diagnoses was most strongly associated with worse mood scores, with a smaller, but significant, association with lower serum TT concentrations. Several studies have reported higher rates of mood disorders in men with current, or prior, history of androgen abuse compared with men without; however, it is not known whether this is a preexisting risk factor for androgen abuse or caused by it (16, 47, 48). It has been suggested that men who experience greater social physique anxiety report more severe androgen dependence and greater psychological adverse effects, while impaired risk perception and continued substance use despite known harms are recognized features of substance dependence and may also contribute to psychiatric symptoms (49, 50). While supraphysiological androgen exposure does not act via classical reward receptors, animal studies have demonstrated alterations in opioid signaling, including increased β-endorphin activity and changes in opioid receptor expression (51, 52). Concurrent illicit substance use is common among individuals who abuse androgens and may modify or exert additive effects on neural reward pathways, altering psychological symptoms. Neuroimaging studies in humans have also identified structural and functional alterations in the limbic regions, including the amygdala, which may contribute to impaired impulse control, dependence-related behaviors, and withdrawal symptoms (53, 54). In contrast to our findings, other studies have not demonstrated an association between serum TT concentration and mood in men with a history of androgen abuse; however, many of these cohort comprise of men who had ceased used many years prior, suggesting that this association may weaken over time (18). Not all studies however report high rates of mood disturbance with androgen abuse. Smit et al (55) found no differences in mild or moderate depression during or after androgen abuse; however, they had very few men at baseline with evidence of depression (6% mild and 4% moderate), which is lower than would be anticipated in an androgen abusing cohort.

Our findings, in alignment with other studies, suggest that the mood symptoms experienced with cessation may be a worsening, or reemergence of preexisting psychological symptoms which were not addressed, or only partially treated, prior to androgen abuse. The association between lower TT concentrations and poorer mood scores raises the possibility that pharmacological interventions with testosterone or related agents could have a role in managing androgen abuse withdrawal. However, this requires further high-quality randomized controlled trials before being incorporated into clinical practice. In other populations, particularly middle-aged and older men, low serum TT has been associated with fatigue and depressive symptoms, and testosterone treatment has been shown to produce modest improvements in mood (56).

We report that sexual dysfunction, as measured by the IIEF-15, was worse in men who had stopped androgen abuse compared with nonusers or current users. In men with organic hypogonadism, low testosterone concentrations are unequivocally associated with worse sexual function (42, 57). In the EMAS study, TT thresholds were associated with specific sexual symptoms: <11 nmol/L with poor morning erections, <8 nmol/L with low frequency of sexual thoughts, and <8.5 nmol/L with erectile dysfunction (58). We were surprised to observe that while there was a mild positive correlation between TT concentration and sexual function in our whole study cohort, there was no independent association with TT concentration and IIEF-15 total scores on multivariable linear regression. This reflects that male sexual function is multidimensional and is influenced by hormonal, physical and psychological factors.

Impairment in sexual function is commonly reported by men with a history of androgen abuse. In a large meta-analysis of 2411 participants, 31% reported decreased libido and 19% reported erectile dysfunction, with other cross-sectional studies reporting similar findings (16, 59, 60). Both Rasmussen et al (13) and Kanayama et al (14) described worse sexual function in men with a prior history of androgen abuse but found no association between these symptoms and serum testosterone levels, similar to our results. In contrast to our study however, the mean time since cessation was 2.5 and 10 years, respectively. Our data suggests that sexual dysfunction occurs shortly after cessation, and despite 60% of men having a serum TT concentration of above 12 nmol/L, it is likely this symptom will persist for many years without intervention. In our multivariable analysis, increasing age was significantly associated with worse sexual function, with each 10-year increment in age corresponding to an 8-point reduction in IIEF-15 score. Previous studies have demonstrated age-related declines in sexual function in men, which may be attributable to declines in testosterone concentrations and/or the accumulation of comorbidities such as obesity, diabetes, and vascular disease, conditions known to directly impair sexual function (58, 61, 62).

Unexpectedly, we observed an independent association between serum LH concentrations and IIEF-15 total scores. The relationship was nonlinear, with LH values below 4 IU/L showing no association, but above this threshold, higher LH levels were progressively associated with lower IIEF-15 scores. Previous cross-sectional studies have demonstrated reduced testicular volume with current androgen abuse, which can persist after cessation (13). More recently, Bulut et al (63) reported lower INSL3 concentrations and a blunted testosterone response to hCG stimulation in former users, consistent with impaired Leydig cell capacity, with a longer cumulative duration of androgen use associated with greater impairment. We hypothesize that the observed inverse association between LH and sexual function above a certain threshold may reflect early Leydig cell dysfunction in this population. Although INSL3 and hCG stimulation testing was not performed in our participants, the use of high-sensitivity urinary testing allowed us to exclude ongoing androgen use or testosterone-stimulating drugs, which may have falsely altered LH levels. Further studies are required in the future to clarify the true clinical significance of this finding.

We report that QOL scores, across many domains, are lowest in the group with a history of androgen abuse, compared with current or nonusers. Similarly to other symptoms, we found that a self-reported psychiatric diagnosis was the stronger independent predictor of these symptoms, while testosterone concentrations and other features of use were not associated. A fear of reduced QOL is one of the biggest barriers to cessation (35). Bulut et al (17) performed a similar cross-sectional study only examining SF-36 scores as a marker of QOL in men with current, former or no androgen abuse. Like our study, men with a history of prior androgen abuse had significantly lower scores in many of the SF-36 domains; however, they found that lower serum TT concentrations were a determinant of the mental component score. While the past-using groups had similar TT levels in both studies, our cohort had a much higher percentage of psychiatric diagnoses as they excluded men with severe psychiatric disease. Our cohort is therefore more reflective of the true androgen abuse cohort, as psychiatric diagnoses are common and frequently severe. In addition, their study had a longer time elapsed since androgen cessation (2.5 years vs 5 months) compared with our study. The difference in findings may also reflect geographical differences between Denmark and the United Kingdom, particularly in substance misuse rates, burden of mental health problems, and country-specific harm reduction strategies and support. While Bulut et al (17) show that QOL is prolonged and persistent in men with a history of androgen abuse, our findings show that this occurs within a few months of cessation and highlights the need for timely intervention. However not all studies have shown impaired QOL; Smit et al (55) reported no reduction in QOL scores using the World Health Organization QOL scale, around 1 year after cessation; however, the duration of use was much shorter and number of those with a psychiatric condition at baseline much lower (55).

## Conclusion

In conclusion, men within 1 year of androgen abuse cessation experienced significantly worse mood, anxiety, sexual function, and QOL compared with both nonusers and current users. Psychiatric comorbidity was the strongest determinant of symptoms severity, with biochemical markers of HPG axis recovery showing a smaller association. These findings challenge the hypothesis that androgen withdrawal symptoms are predominantly driven by hypogonadism and highlight the importance of psychological factors. These results support the need for accessible and effective multidisciplinary services for individuals seeking support following cessation and the evaluation of psychological and behavioral interventions, rather than pharmacological ones, in supportive androgen withdrawal therapies for men.

## Data Availability

Some or all datasets generated during and/or analyzed during the current study are not publicly available but are available from the corresponding author on reasonable request.
